# Chimeric antigen receptor T cell therapy: a new emerging landscape in autoimmune rheumatic diseases

**DOI:** 10.1093/rheumatology/kead616

**Published:** 2023-11-20

**Authors:** Xia Lyu, Latika Gupta, Eleni Tholouli, Hector Chinoy

**Affiliations:** Department of Rheumatology, Renji Hospital, Shanghai Jiaotong University School of Medicine, Shanghai, China; Epidemiology and Public Health Group, School of Health Sciences, The University of Manchester, Manchester, UK; Division of Musculoskeletal and Dermatological Sciences, Faculty of Biology, Medicine and Health, The University of Manchester, Manchester, UK; Division of Musculoskeletal and Dermatological Sciences, Faculty of Biology, Medicine and Health, The University of Manchester, Manchester, UK; Department of Rheumatology, Royal Wolverhampton Hospitals NHS Trust, Wolverhampton, UK; Department of Haematology, Manchester Royal Infirmary, Manchester, UK; Division of Musculoskeletal and Dermatological Sciences, Faculty of Biology, Medicine and Health, The University of Manchester, Manchester, UK; Department of Rheumatology, Salford Royal Hospital, Northern Care Alliance NHS Foundation Trust, Manchester Academic Health Science Centre, Salford, UK

**Keywords:** CAR-T, immunotherapy, autoimmune rheumatic diseases, lupus, myositis, scleroderma

## Abstract

Chimeric antigen receptor T cell (CAR-T) therapy, an innovative immune cell therapy, has revolutionized the treatment landscape of haematological malignancies. The past 2 years has witnessed the successful application of CD19-targeting CAR constructs in refractory cases of autoimmune rheumatic diseases, including systemic lupus erythematosus, systemic sclerosis and anti-synthetase syndrome. In comparison with existing B cell depletion therapies, targeting CD19 has demonstrated a more rapid and profound therapeutic effect, enabling drug-free remission with manageable adverse events. These promising results necessitate validation through long-term, large-sample randomized controlled studies. Corroborating the role of CAR-T therapy in refractory rheumatological disorders and affirming safety, efficacy and durability of responses are the aims of future clinical studies. Optimizing the engineering strategies and better patient selection are also critical to further refining the successful clinical implementation of CAR-T therapy.

Rheumatology key messages

- CAR-T therapy can be effective in the management of refractory rheumatic diseases.
- The principles of CAR-T therapy are founded on precise and specific actions against disease-related targets.
- Further clinical trials are needed to validate the efficacy and safety of CAR-T therapy.

## Introduction

Chimeric antigen receptor T cell (CAR-T) therapies have gained recognition as potentially curative treatments in patients with end-stage haematological cancers. An adoptive cell immunotherapy approach utilizes viral transduction or gene editing to modify T lymphocytes and engineer synthetic receptors on their surface. These receptors, known as CARs, possess an extracellular domain that can specifically bind to antigens, and a variable intracellular co-stimulatory domain that can trigger *in vivo* CAR-T expansion and persistence, leading to effective elimination of target tumour cells. CAR-T associated toxicities such as cytokine release syndrome (CRS), neurotoxicity [1] and prolonged B cell aplasia are well recognized. Treatment efficacy and refinement of CAR constructs has enabled safe delivery across patients with a wide breadth of diagnoses. Exploration of CAR-Ts in solid tumours, autoimmune and degenerative diseases and in earlier treatment pathways is underway. The successful application of CD19-targeted CAR T cells in refractory cases of autoimmune rheumatic diseases, including SLE, SSc and anti-synthetase syndrome (ASyS), offers a promising innovative treatment modality. In comparison with existing B cell depletion therapies, targeting CD19 has demonstrated a more rapid and profound therapeutic effect, enabling drug-free remission with manageable adverse events reported.

## CAR-T therapy and its approval in cancer

**Figure 1.**
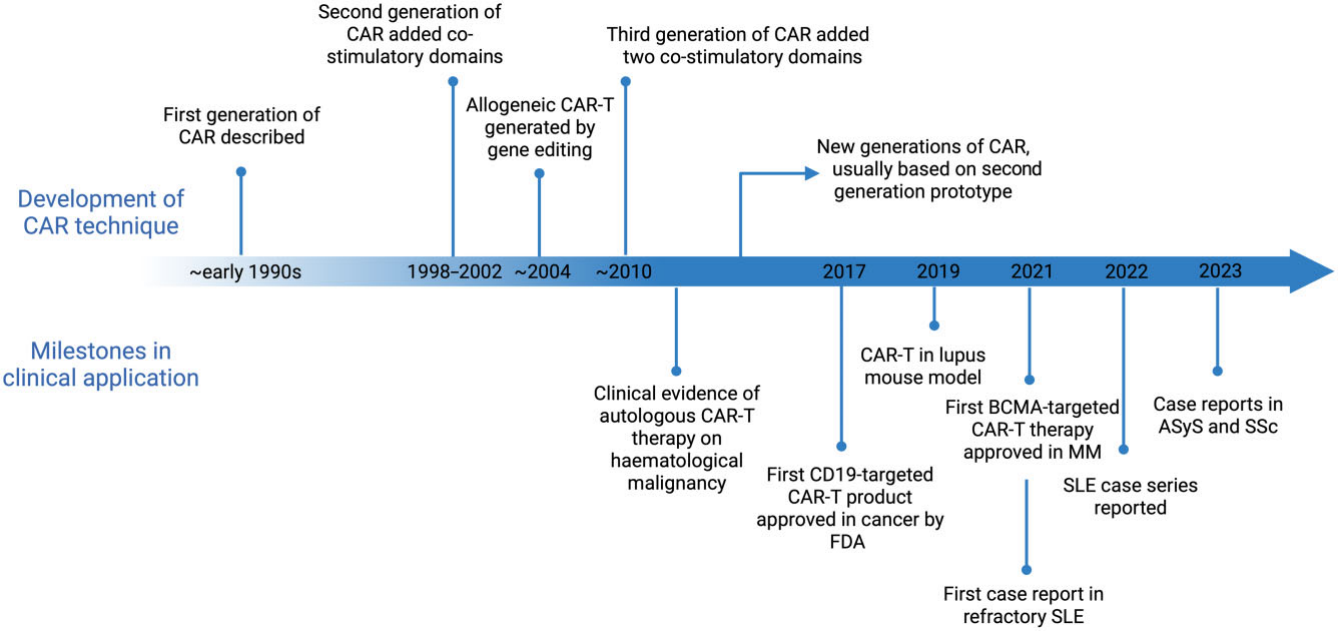
History of chimeric antigen receptor (CAR) immune cell therapy. First constructed around early 1990s, CAR has gone through several iterations. CAR-T therapy was originally designed to target and kill tumour cells. In 2017, the first CD19-targeted CAR-T product was approved in haematological malignancy. The first case of refractory SLE successfully treated with CD19-targeted CAR-T therapy was reported in 2021, after which case reports of CAR-T therapy in autoimmune rheumatic diseases have continued to emerge. ASyS: anti-synthetase syndrome; CAR-T: chimeric antigen receptor T cell; FDA: US Food and Drug Administration. (Image created with BioRender.com)

**Figure 2.**
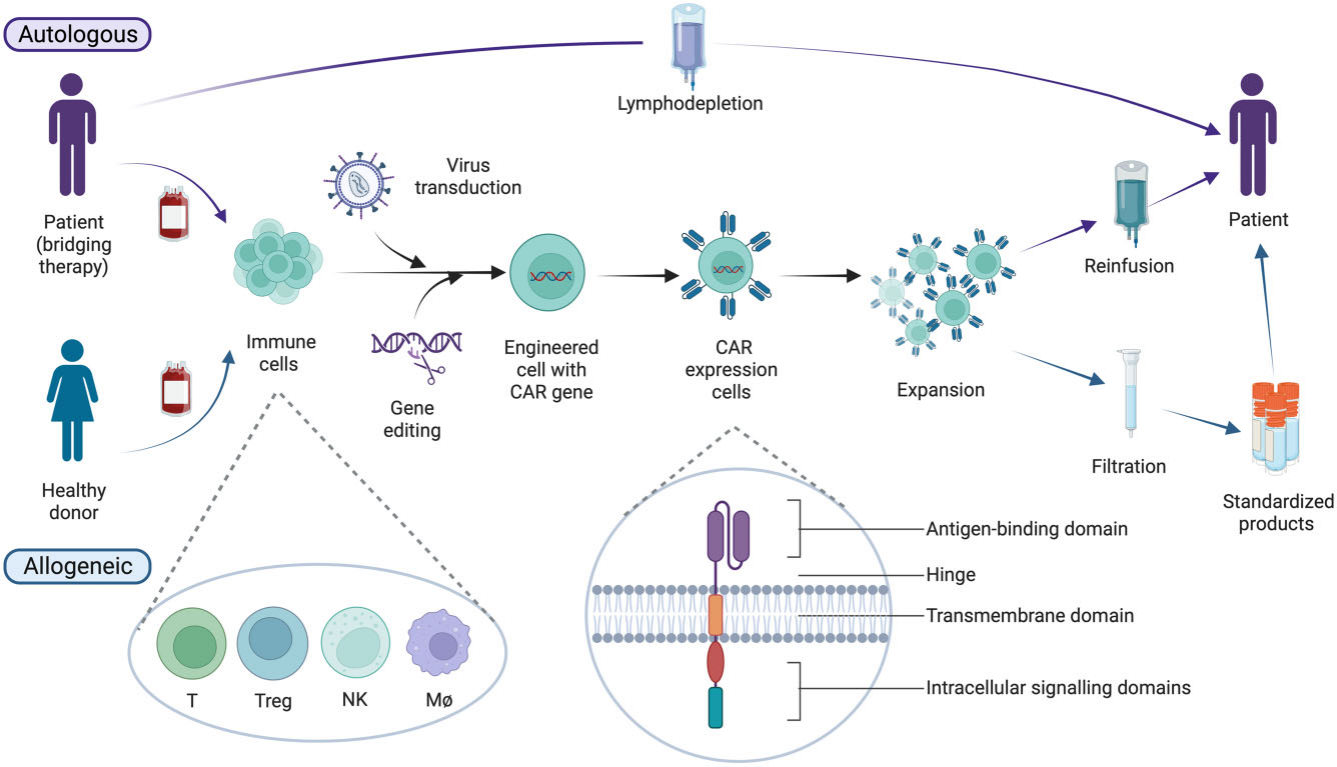
Procedure for chimeric antigen receptor (CAR)-based immune cell therapy. The original cell source can be either autologous (derived from circulating immune cells) or allogeneic (which can also be umbilical cord blood-derived). Although only CAR T cells have been reported in autoimmune rheumatic diseases, engineered Treg cells, NK cells or macrophages are promising as well. CAR genes can be intergrated into target cells through virus transduction and gene editing. The prototypic CAR contains the following parts: an antigen-binding domain, usually scFv, but it can also be designed as an antibody-receptor; a hinge and transmembrane domain; and intracellular signalling domains, usually consisting of different co-stimulatory domains and CD3ζ. After *ex vivo* amplification, CAR immune cells generated by the autologous route can be reinfused into the same patient. In the allogeneic route, CAR immune cells should be filtered to remove the TCR positive cells and stored. In most cases currently, the patient must be pretreated with lymphodepletion conditioning. (Image created with BioRender.com)

CAR T cells were first constructed by Eshhar and colleagues in 1989 (Fig. 1), aiming at expressing functional chimeric T cell receptors (TCR) that recognize antigens in a non-major histocompatibility complex (MHC)-restricted manner [2]. The first generation of CARs consisted of a single-chain variable fragment (scFv) derived from an antibody as the extracellular antigen-binding domain, a hinge or spacer, a transmembrane domain, and an intracellular signalling domain, CD3ζ, from a TCR–CD3 complex [3] (Fig. 2). To increase the proliferative capacity, persistence and cytokine-secreting function of CAR T cells, the second generation included an intracellular co-stimulatory domain, e.g. CD28 or 4-1BB. CAR-edited T cells can specifically recognize and bind to targeted tumour cells. Activated intracellular domains further strengthen the immune response and targeting of tumour cells.

## Efficacy, tolerance and safety of CAR-T therapy

The efficacy and tolerance of CAR T cells was proven in early phase clinical trials, and they were approved for clinical use in 2017 (Fig. 1) [4, 5]. By July 2023, six CAR-T products were licensed by the European Medicines Agency and the US Food and Drug Administration (FDA). These include CD19-targeting CAR T cells for relapsed refractory large B cell lymphoma (LBCL), follicular lymphoma, mantle cell lymphoma (MCL) and acute lymphoblastic leukaemia (ALL), and B cell maturation antigen (BCMA)-targeting products for relapsed refractory multiple myeloma [6, 7]. Currently approved therapies use autologous T cells, which are collected through leukapheresis. The manufacturing process is complex and takes ∼3–4 weeks. T cells are first edited to express CARs through virus transduction. Synthetic CAR T cells are then expanded *in vitro* and cryopreserved for transport and storage. Prior to CAR-T infusion, patients undergo preparative lymphodepleting chemotherapy [8]. Early CAR-T-related toxicities including CRS and immune effector cell associated neurotoxicity syndrome (ICANS) are product and disease dependent and typically occur within 30 days of infusion, during which period patients are carefully monitored [1, 4, 5]. Tumour-lysis syndrome, neutropenic fevers and other chemotherapy-related toxicities may also occur along with CAR-T specific late toxicities such as prolonged cytopenia, B cell aplasia, hypogammaglobulinaemia, and in rare cases, delayed CRS/ICANS [4, 5].

CRS is a multisystem inflammatory response to CAR T cell activation and expansion that can mimic sepsis. This cytokine storm typically results in fevers with or without hypoxia and/or hypotension, sometimes requiring critical care intervention. In rare cases, haemolymphophagocytic/macrophage activation syndrome can develop. The pathophysiology of ICANS is less well understood. Symptoms can range from mild tremor, poor concentration and lethargy to expressive aphasia, confusion and drowsiness. Patients may develop stroke-like symptoms, seizures and occasionally cerebral oedema. Baseline observations as well as neurological assessments are performed multiple times throughout a 24-h period. The latter includes an extended mini-mental state assessment, the Immune-effector Cell Encephalopathy (ICE) score, which evaluates the patients’ orientation, handwriting, and ability to follow commands, name items and count. Published consensus guidelines by the American Society for Transplantation and Cellular Therapy are followed to grade these toxicities [1] (Table 1). Treatment algorithms endorsed by the European Society for Blood and Bone Marrow Transplantation [9], the European Haematology Association [8] and the American Society of Clinical Oncology [10] include supportive care with fluid replacement, oxygen supplementation and vasopressors as indicated, treatment of potentially underlying infections, as well as targeted therapies such as use of tocilizimab for CRS. Steroids can be effective for both CRS and ICANS, and in refractory or severe cases are combined with other immune modulators, e.g. anakinra (IL-1 receptor antagonist) and siltuximab (monoclonal anti-IL-6).

**Table 1.** ASTCT consensus grading^a^ for cytokine release syndrome (CRS) and immune effector cells associated neurotoxicity syndrome (ICANS)

	Grade 1	Grade 2	Grade 3	Grade 4
CRS grading; parameter^b^				
Fever (temperature ≥38°C)	Yes	Yes	Yes	Yes
with hypotension	None	Not requiring vasopressors	Requiring a vasopressor with or without vasopressin	Requiring multiple vasopressors (excluding vasopressin)
and/or hypoxia	None	Requiring low-flow nasal cannula or blow-by	Requiring high-flow nasal cannula, facemask, non-rebreather mask, or Venturi mask	Requiring positive pressure
ICANS grading; neurotoxicity domain^b^				
ICE score	7–9	3–6	0–2	0 (patient is unarousable and unable to perform ICE)
Depressed level of consciousness	Awakens spontaneously	Awakens to voice	Awakens only to tactile stimulus	Patient is unarousable or requires vigorous or repetitive tactile stimuli to arouse. Stupor or coma
Seizures	N/A	N/A	Any clinical seizure focal or generalized that resolves rapidly or non-convulsive seizures on EEG that resolve with intervention	Life-threatening prolonged seizure (>5 min); or repetitive clinical or electrical seizures without return to baseline in between
Motor findings	N/A	N/A	N/A	Deep focal motor weakness such as hemiparesis or paraparesis
Elevated ICP/cerebral oedema	N/A	N/A	Focal/local oedema on neuroimaging	Diffuse cerebral oedema on neuroimaging; decerebrate or decorticate posturing; or cranial nerve VI palsy; or papilledema; or Cushing's triad

a See the original guideline for more details [1].

b Both CRS and ICANS grade are determined by the most severe event not attributable to any other cause. ICE: Immune Effector Cell-Associated Encephalopathy; ICP: intracranial pressure; N/A: not applicable.

The largest UK experience to date is with axicabtagene (Yescarta^®^) and tisagenlecleucel (Kymriah^®^), fast-tracked and commissioned by NHS England for relapsed refractory LBCL [11] and paediatric ALL in 2019, and brexucabtagene (Tecartus^®^) for MCL in 2021 and adult ALL in 2023. The overall incidence of CRS reported is up to 95% across all grades for all products and indications, with grades 3–4 occurring in 5–13% of patients treated with axicabtagene or tisagenlecleucel for LBCL [12–16], 15% treated with brexucabtagene for MCL [17, 18], and 30% treated with brexucabtagene for ALL [19]. The median time to onset was 2–4 days post-infusion (range 1–15) and the median duration 6–10 days (range 1–43). The incidence of all grade ICANS is 30% for tisagenlecleucel of which up to 10% are grades 3–4, and 60–80% for the other products with 30–40% attributed to grades 3–4. Severity of both CRS and ICANS is believed to be related to disease bulk, but most patients experience a full recovery with a treatment-related mortality in this patient population of <5% [4].

## Clinical reports of CD19-targeted CAR-T therapy in autoimmune rheumatic diseases

The successful application of CAR-T therapy in haematological malignancies has inspired its use in refractory autoimmune rheumatic diseases (ARDs) [20]. Preclinical data suggested compelling evidence that CD19-targeted CAR CD8^+^ T cells possess the capability to effectively deplete CD19^+^ B cells, leading to the elimination of autoantibody production and subsequent reversal of lupus manifestations in both NZB/W F_1_ and MRL/lpr mice models [21]. Additional clinical evidence has been reported as outlined in Table 2.

**Table 2.** Summary of reports on the application of CAR-T therapy in refractory autoimmune rheumatic diseases^a^

Disease	Patients, gender/age	Previous treatment	Lymphodepletion	Intervention	Dose (CAR T cells/kg body weight)	AEs and treatment	Follow-up time	Drug-free time	Reference
SLE	F/20	High-dose glucocorticoids, HCQ, MMF, TAC, RTX and BEM	Fludarabine and cyclophosphamide	Autologous CD19-targeted CAR T cells	1.1 × 10^6^	None	7w	0d (low-dose glucocorticoid)	[22]
SLE	F/22, M/23, F/22, F/24, F/18	Pulsed steroids, HCQ, MTX, LEF, AZA, MMF, CTX, TAC, RTX or BEM	Fludarabine and cyclophosphamide	Autologous CD19-targeted CAR T cells	1 × 10^6^	3/5 had CRS (Grade 1), 3 received metamizole and 1 received TOC	22m/16m/14m/13m/11m	22m/16m/14m/13m/11m	[23, 24]
SLE	F/33, F/33	No data	Fludarabine and cyclophosphamide (50% dose-reduced for the second patient)	Autologous CD19-targeted CAR T cells	1 × 10^6^	CRS (Grade 1)	8m/4m	8m/4m	[24]
SSc	M/60	MTX and MMF	Fludarabine and cyclophosphamide (50% dose-reduced due to renal impairment)	Autologous CD19-targeted CAR T cells	1 × 10^6^	CRS (Grade 1)	6m	6m	[25]
ASyS (Jo-1)	M/41	Glucocorticoids, CTX, TAC, RTX, and IVIG	Fludarabine and cyclophosphamide	Autologous CD19-targeted CAR T cells	1 × 10^6^	CRS (Grade 1), paracetamol and TOC	180d	180d	[26]
ASyS (Jo-1)	M/41	Glucocorticoids, MTX, LEF, BARI, RTX, AZA and IVIG	Fludarabine and cyclophosphamide	Autologous CD19-targeted CAR T cells	1.23 × 10^6^	CRS (Grade 1)	240d	28d (AZA/MMF)	[27]
ASyS (Jo-1)	F/44	Glucocorticoids, CTX, MMF, TAC, TOF, TOC, IVIG, RTX, OC and LEN	Fludarabine and cyclophosphamide	Autologous CD19-targeted CAR T cells	1 × 10^6^	CRS (Grade 1), TOC; ICANS (Grade 1), DEX	150d	150d	[28]

a Only cases with detailed clinical information included. ASyS: anti-synthetase syndrome; AZA: azathioprine; BARI: baricitinib; BEM: belimumab; d: days; DEX: dexamethasone; CRS: cytokine release syndrome; CTX: cyclophosphamide; F: female; HCQ: hydroxychloroquine; ICANS, immune-related effector cell neurotoxicity syndrome; IVIG: immunoglobulins; LEF: leflunomide; LEN: lenabasum; m: months; M: male; MMF: mycophenolate mofetil; MTX: methotrexate; OC: ocrelizumab; RTX: rituximab; TAC: tacrolimus; TOC: tocilizumab; TOFA: tofacitinib; w: weeks.

Mougiakakos *et al*. first reported the use of autologous CD19-targeted CAR T cells in a 20-year-old female with refractory SLE [22]. The patient had active lupus nephritis, pericarditis, endocarditis, pleurisy, rash and arthritis, and failed treatment with high-dose glucocorticoids and multiple immunsuppressants previously, including two B cell-targeting biologics, rituximab and belimumab. After treatment with fludarabine (25 mg/m^2^/d intravenously [i.v.] from day −5 to day −3) and cyclophosphamide (1000 mg/m^2^/d i.v. on day −3), the patient received CAR T cells at a dose of 1.1 × 10^6^ cells/kg body weight once, and experienced a rapid remission within 44 days, not only serologically with anti-double-strand DNA (dsDNA) antibodies turned negative and low completement C3 and C4 levels normalized, but also clinically with urine protein from above 2000 mg/g to <250 mg/g and SLEDAI-SELENA score from 16 to 0. No adverse events were observed within a 7-week follow-up. Low-dose prednisolone was the sole remaining medication since lymphodepletion, and it was successfully discontinued without any indications of disease relapse for a period of up to 18 months [29].

The same team further reported a group of five successful SLE cases with CD19-targeted CAR-T platform [23]. The patients were between 18 and 24 years old with at least 1 year disease duration, previously treated with pulse glucocorticoids, hydroxychloroquine, mycophenolate, cyclophosphamide and other immunosuppressive drugs. Notably, all five patients received belimumab and one used rituximab. All of them had multiorgan involvement with histology-proven glomerulonephritis, and had active disease at baseline with SLEDAI-2K scores between 8 and 16. The procedures were similar to the first case, but these patients were totally drug free since CAR-T therapy and received a slightly low dose at 1.0 × 10^6^ cells/kg body weight. SLEDAI-2K scores, proteinuria, completement C3 levels and anti-dsDNA antibody levels showed a significant improvement within 3 months, as well as patient self-rated fatigue. Circulating B cell numbers fully disappeared from day 2 and remained zero for a prolonged period, while other circulating immune cells recovered within 10 days after treatment. The observed adverse events were limited to Grade 1 CRS in three out of five patients. These Grade 1 CRS cases were effectively managed. All five patients remained drug-free and no SLE flare occurred during follow-up [24]. In summary, the autologous CD19-targeted CAR-T therapy demonstrated both efficacy and tolerability in active SLE patients, as evidenced by relatively long-term follow-up.

CAR-T therapy has also been reported in severe and refractory SSc [25]. In one instance, a 60-year-old male with diffuse cutaneous SSc, presenting with anti-RNA polymerase III autoantibodies and multiple organ involvement, underwent CD19-targeted CAR-T therapy at a dosage of 1.0 × 10^6^ cells/kg body weight. Due to renal impairment, the patient received a reduced dose of fludarabine and cyclophosphamide for lymphodepletion conditioning. Prior to the therapy, the patient exhibited various disease manifestations, including MRI-confirmed diffuse myocardial fibrosis, high-resolution CT-verified lung fibrosis, right heart catheterization-validated pulmonary hypertension, Raynaud’s phenomenon and carpal arthritis. Despite prior treatment with methotrexate and mycophenolate, the patient experienced treatment failure. Following the CAR-T treatment, there was a noteworthy turnaround in the patient's condition, evidenced by the reversal of anti-RNA polymerase III autoantibodies and significant improvement in heart, lung, joint and skin manifestations at both 3 and 6 months' follow-up. Notably, Grade 1 cytokine-release syndrome was observed, although the administration of tocilizumab was not necessary for management. In comparison with previously reported SLE cases, the patient in this instance was older, further highlighting the remarkable clinical efficacy and tolerability of CD19-targeted CAR-T therapy in the context of severe and refractory systemic sclerosis.

Similar applications have been reported in refractory ASyS, a systemic rheumatic disease manifested as positive anti-synthetase antibody, myositis, interstitial lung disease (ILD), fever, arthritis, mechanic's hands and Raynaud's phenomenon. Two 41-year-old male ASyS patients with anti-Jo-1 antibodies experienced significant improvement and sustained stability following CD19-targeted CAR-T therapy [26, 27]. Both patients failed with mutiple immunosuppressants, including rituximab and intravenous immunoglobulins, and presented with severe muscle weakness and progressive shortness of breath at baseline. After CAR-T treatment, both patients showed rapid clinical improvement with negative anti-Jo-1 antibody, decreased creatine kinase level, improved manual muscle test score, and improved Physician Global Assessment score. Although similar, there were differences between these two cases, including the dose of CAR T cells, the dose of glucocorticoids before conditioning, the speed of B cell recovery, and the overall follow-up time. One patient remained drug-free for 180 days, while the other received additional azathioprine (changed to mycophenolate mofetil later) 28 days after CAR T cell infusion. Both patients experienced Grade 1 CRS, and only one patient received tocilizumab.

A further report outlines the course of a 44-year-old lady with ASyS with positive anti-Jo-1, anti-Pm-Scl-100 antibodies and high-level rheumatoid factor [28]. The patient experienced refractory myositis and arthritis, as well as uncontrolled lung and skin manifestation. She failed on glucocorticoids, cyclophosphamide, mycophenolate, tacrolimus, tofacitinib, tocilizumab, IVIG, rituximab and ocrelizumab, and showed only slight improvement with lenabasum in a clinical trial. Following CD19-targeted CAR-T therapy, a remarkable improvement was noted in muscle, joint and lung conditions, achieving major improvement according to the 2016 ACR/EULAR Total Improvement Score. Moreover, the patient maintained drug-free status during the 150-day follow-up. While experiencing mild CRS, the patient developed mild dizziness 1 week after CAR-T infusion, raising concern for ICANS. Dexamethasone administration promptly resolved the symptoms.

An increasing number of cases are continuing to emerge. In the most recent reports, as of June 2023, the Erlangen (Germany) team has administered CD19-targeted CAR-T therapy to eight patients with treatment-resistant SLE, with the longest follow-up period extending to 24 months [30], as well as three idiopathic inflammatory myopathy patients and four SSc patients [31]. Another three patients with severe refractory SLE have been reported recently as a sentinel cohort enrolled in an open-label, single-arm, multicentre phase 1/2 study (NCT05798117, Table 3) [32]. Notably, these patients are older compared with SLE patients previously reported, with ages of 58, 48 and 38, respectively. Although the longest follow-up period of these three cases is 106 days, the disclosed data indicate initial efficacy and manageable tolerability.

**Table 3.** CAR therapy studies in autoimmune rheumatic diseases registered on ClinicalTrials.gov, by 30 OCT 2023

NCT number	Study status	Condition	Conditions other than ARD	Interventions	Cellular resource	Targeting	Cell type	Age	Phase	Start date	Last update posted	Country
NCT04561557	Recruiting	IMNM	NMOSD/MG/CIDP	CT103A cells	Autologous	BCMA	T	Adult, older adult	Early phase 1	22/09/2020	12/04/2023	China
NCT05030779	Unknown	SLE		CD19/BCMA CAR T cells	NA	BCMA+CD19	T	Child, adult, older adult	Early phase 1	10/09/2021	01/09/2021	China
NCT05085418	Recruiting	SLE (LN)		CD19/BCMA CAR T cells	NA	BCMA+CD19	T	Child, adult, older adult	Early phase 1	05/11/2021	20/10/2021	China
NCT05085431	Recruiting	SS		CD19/BCMA CAR T cells	NA	BCMA+CD19	T	Child, adult, older adult	Early phase 1	05/11/2021	20/10/2021	China
NCT05085444	Recruiting	SSc		CD19/BCMA CAR T cells	NA	BCMA+CD19	T	Child, adult, older adult	Early phase 1	08/10/2021	20/10/2021	China
NCT05239702	Recruiting	DM/Still’s disease	CD/UC	CD7 CAR T cells	NA	CD7	T	Child, adult, older adult	Early phase 1	28/02/2022	15/02/2022	China
NCT05263817	Recruiting	Vasculitis	POEMS/Amyloidosis/AIHA	CD19/BCMA CAR T cells	NA	BCMA+CD19	T	Child, adult, older adult	Early phase 1	08/10/2021	03/03/2022	China
NCT05459870	Recruiting	AD (not specified)		4SCAR T cells	NA	CD19/BCMA/CD138/BAFF-R	T	Adult, older adult	Phase 1/phase 2	31/07/2022	19/07/2022	China
NCT05474885	Recruiting	SLE		BCMA-CD19 CAR T cells	Autologous	BCMA+CD19	T	Adult, older adult	Phase 1	01/04/2022	26/07/2022	China
NCT05765006	Recruiting	SLE		Relma-cel	Autologous	CD19	T	Adult, older adult	Phase 1	24/02/2023	15/03/2023	China
NCT05798117	Recruiting	SLE		YTB323	Autologous	CD19	T	Adult, older adult	Phase 1/phase 2	28/02/2023	28/09/2023	France, Germany, Spain
NCT05846347	Recruiting	SLE		GC012F	Autologous	BCMA+CD19	T	Adult, older adult	Phase 1	15/05/2023	11/05/2023	China
NCT05858684	Recruiting	SLE		GC012F	Autologous	BCMA+CD19	T	Adult, older adult	Early phase 1	11/05/2023	15/05/2023	China
NCT05859997	Recruiting	SLE/SS/dcSSc/IIM/AAV/APS		BRL-301	Allogeneic	CD19	T	Adult, older adult	NA	17/05/2023	18/10/2023	China
NCT05869955	Recruiting	SLE		CC-97540	Autologous	CD19	T	Adult, older adult	Phase 1	13/09/2023	18/10/2023	USA
NCT05930314	Enrolling by invitation	SLE (LN/ITP)		CNCT19 cell	Autologous	CD19	T	Adult, older adult	Early phase 1	19/06/2023	05/07/2023	CN
NCT05938725	Recruiting	SLE (LN)		KYV-101	Autologous	CD19	T	Adult, older adult	Phase 1	28/04/2023	10/07/2023	USA

Definition of age groups: child (birth–17 years); adult (18–64 years); older adult (65+ years). AAV: ANCA-associated vasculitis; AD: atopic dermatitis; AIHA: autoimmune heamolytic anemia; ARD: autoimmune rheumatic disease; BAFF-R: B cell activating factor-receptor; BCMA: B cell maturation antigen; CIDP: chronic inflammatory demyelinating polyneuropathy; CD: Crohn's disease; IIM: idiopathic inflammatory myopathy; IMNM: immune-mediated necrotizing myopathy; MG: myasthenia gravis; NMOSD: neuromyelitis optica spectrum disorder; NA: not applicable; POEMS: polyneuropathy, organomegaly, endocrinopathy, monoclonal gammopathy, and skin changes syndrome; UC: ulcerative colitis.

## CAR-T therapy for malignancy comorbid with autoimmune rheumatic diseases

A topic worthy of attention is CAR-T therapy in the treatment of patients with previously diagnosed ARDs who develop haematological malignancies. Patients with autoimmune diseases have an increased risk of developing lymphoproliferative malignancy, especially large B cell, marginal zone and T cell subtypes [33, 34]. However, previous clincial trials of CAR-T therapy for B cell lymphoma have excluded patients with history of autoimmune disorders and those on immunosuppressive medications or steroids.

Zhang *et al.* reported a case in which a lymphoma patient with long-term SLE benefited from CAR-T therapy for both diseases in 2021 [35]. The 41-year-old female, who was diagnosed with SLE 20 years ago, developed diffuse large B cell lymphoma (DLBCL), and was enrolled in a clinical trial using BCMA/CD19 dual-targeted CAR-T therapy due to side effects of standard R-CHOP chemotherapy. Pretreated with fludarabine and cyclophosphamide and with discontinued maintenance prednisone, she received a single infusion of CAR T cells at a dose of 5.3 × 10^6^/kg body weight. She experienced a remarkable remission of DLBCL, accompanied by negative seroconversion of autoantibodies. The patient had B cell aplasia persisting for 198 days, and maintained a drug-free remission throughout the 23-month follow-up period.

Based on a large-scale electronic health records database in the USA, Wang *et al.* recently reported on 1363 adult patients diagnosed with B cell non-Hodgkin lymphoma (NHL) and treated with FDA-approved CAR-T therapy from 2019 and 2023 [36]. Among them, 58 cases (4.3%) had concomitant ARDs, including 24 RA, 13 psoriasis, 10 SLE, five Sjögren's syndrome, four ankylosing spondylitis and two polymyalgia rheumatica. Most patients were on immunosuppressants at baseline, including hydroxychloroquine, methotrexate, mycophenolate and tacrolimus. An additional 58 non-autoimmune patients were selected through propensity score matching, and time to next treatment or death and overall survival showed no significant difference between the two groups. In terms of safety, there was no significant difference regarding CRS, ICANS, or medications for adverse events. Thirteen (31%) patients received prednisone, and five patients restarted hydroxychloroquine and methotrexate within 1 year after CAR-T treatment. Although limited by its retrospective nature, this study provides meaningful evidence for the use of CAR-T therapy in patients with concurrent ARD and B cell NHL [37].

## Advantages over existing immunosuppresive therapies and main concerns

The published ARD cases all involved refractory conditions with significant organ involvement. Prior to undergoing CAR-T therapy, these patients had been treated with various immunosuppressive drugs, but none of them achieved long-term remission or low disease activity. In some instances, B cell-targeted therapies, such as B cell depletion using anti-CD20 monoclonal antibodies and monoclonal antibodies targeting B lymphocyte stimulator (BLyS or BAFF), were used, but they either elicited poor responses or resulted in only transient and partial improvements.

B cell lineages play a key role in autoimmune diseases, including producing pathogenic autoantibodies such as anti-dsDNA antibody in SLE, as well as antibody-independent functions like cytokine secreting leading to tissue inflammation directly, or crosstalk with other immune cells triggering immune intolerance [38, 39]. The mechanism of B cells in autoimmune diseases, although not fully elucidated, laid the foundation of B cell depletion therapies [40]. Belimumab, a fully humanized anti-BLyS monoclonal antibody, is the first approved B cell related biologic for ARDs and is currently approved for use in SLE. With numerous preclinical and clinical evidence, anti-CD20 monoclonal antibody like rituximab has been recommended in many guidelines and widely used in clinical management of SLE [41], antiphospholipid syndrome [42], RA [43], antineutrophil cytoplasmic antibody-associated vasculitis [44], SSc [45], IIM [46] and others, especially in severe cases. However, refractory cases with no or little response to rituximab do exist. A possible reason for variable response to B cell related therapies could be incomplete B cell depletion. Compared with CD20, CD19 is more widely expressed during all phases of B cell development, including pro-B cells and antibody-secreting plasmablasts and plasma cells [47]. Targeting CD19 is expected to yield a faster, more extensive and profound B cell depletion effect compared with anti-CD20 monoclonal antibody therapy, including long-lived plasma cells residing in the bone marrow, thus further enhancing the therapeutic potential of CD19-targeted approaches [40].

Drug-free remission was sustained in the follow-up despite the recovery of B cells and immunoglobulins. Notably, in the reported SLE cases, although B cell reconstitution occurred about 100 days after CAR-T infusion, immune phenotyping and B cell receptor sequencing indicated these B cells had a naïve phenotype [23], suggesting that the B cell compartment had been reset successfully [48]. On the other hand, recovered immunoglobulin levels suggested that compared with traditional regimens such as pulse glucocorticoids, CAR-T therapy would not bring comprehensive immunosuppression despite the pre-treatment lymphocyte depletion. Although the experience in cancers shows that disease relapses may occur following CAR T cell infusion, the observed drug-free remission in selected case reports has thus far exceeded expectations. Persistent serological conversion to negative has been reported in SLE cases in follow-up after CAR T therapy, while vaccine-related immune responses are preserved [30]. If remission or low disease activity can be maintained for a longer period, the drug burden of patients will be greatly reduced, including the long-term side effects of glucocorticoids.

Due to the limited extent of preclinical research and clinical reports, several concerns persist regarding the implementation of CAR-T therapy. Two major adverse events, CRS and ICANS, commonly observed in haematological malignancies [26], have also been reported in ARDs. The diverse organ involvements in ARD patients may pose challenges in managing adverse events. For example, fever, a common symptom of CRS, may complicate the management of patients with ILD [31]. Additionally, other considerations involve the waiting time required for *in vitro* engineering and the efficacy of bridging therapies. Disease deterioration has been observed during immunosuppressant washout prior to lymphodepletion therapy [32]. The risk of life-threatening infections due to lymphodepletion pre-processing further adds to safety concerns [27, 28].

B cells might only represent a partial aspect of these diseases, rather than encompassing the entirety of their pathogenesis. The ASyS case mentioned earlier [13] exemplifies this complexity, wherein enhanced CD8^+^ T cells were detected in circulation following CAR-T treatment, leading to the incorporation of additional T cell suppressive drugs due to concerns about potential relapse. A recent report of the failure of murine CD19-targeted CAR-T therapy on an SSc animal model also suggested that CAR-T design should be consistent with disease pathogenesis [49]. The study used a Fra-2 transgenic mouse model, which could well mimic the fibrosis and vasculopathy of SSc but lacks the typical autoimmune response [50, 51]. Although CD19-targeted CAR-T combined with anti-CD20 monoclonal antibody achieved deeper B cell depletion in the peripheral blood and lungs of Fra-2 mice than anti-CD20 monoclonal antibody alone, it did not improve overall outcomes, and even worsened the pulmonary fibrosis and pulmonary hypertension [49]. This is partly because B cells do not play a key role in the pathogenesis of Fra-2 transgenic mice, verified by the result that the anti-CD20 monoclonal antibody group did not show a significant difference compared with the control group. Meanwhile, lack of lymphodepletion pre-processing may lead to the activation of T cells. The report once again confirms the importance of CAR-T design and treatment subject selection.

To fully comprehend the safety and efficacy of this novel therapy, longer follow-up observations and randomized controlled trials are imperative. Moreover, the high cost currently restricts widespread application, particularly considering the potential necessity for repeated treatments. The durability and tolerability of repeated CAR-T therapy in ARDs remain unclear.

## Potentials of CAR-based therapy and ongoing clinical trials

Although the CAR T cells used in the reported ARD cases without malignancy were all CD19-targeted cells generated by lentiviral transduction into autologous T cells, CAR therapy has more diverse designs. We summarize the ongoing CAR-based clinical trials in ARDs in Table 3, and many variant products based on the CAR prototype have been registered for clinical research. Notably, although we focus on rheumatic diseases, the application of CAR-based therapy in other autoimmune diseases has made impressive progress.

The diversity of antigen-binding domains could broaden the targeting field of CAR [52]. In addition to CD19, BCMA, which is almost exclusively expressed on plasmablasts and plasma cells and regulates B cell proliferation, maturation and survival, is a popular target. As mentioned above, BCMA-targeted CAR-T therapy has been approved for the treatment of refractory multiple myeloma, and phase 1 trial interim results of anti-BCMA CAR-T therapy CT103A in relapsed or refractory AQP4-IgG seropositive neuromyelitis optica spectrum disorders have been published [53]. Several clinical trials using BCMA-targeted CAR-T therapy or even BCMA/CD19 dual-targeted CAR-T therapy are planned in refractory ARDs, including SLE, Sjögren's syndrome, immune-mediated necrotizing myopathies, scleroderma and vasculitis (Table 3). Another potential target is CD7, a transmembrane glycoprotein expressed by T cells and NK cells and their precursors. Theoretically, targeting CD7 is more effective than CD19 for autoimmune diseases with abnormal activation of T cells. Anti-CD7 CAR-T therapy has been reported effective in treating T cell malignancies, and a clinical trial in refractory dermatomyositis, Still’s disease and inflammatory bowel diseases is recruiting (NCT05239702). Besides, the extracellular antigen-binding domain of CAR is usually a scFv but can also be designed as antibody-receptors or ligands. Chimeric autoantibody receptor T cells (CAART) provides a more exquisite targeting to cells expressing specific antibodies, which may be more effective in autoimmune diseases caused by pathogenic autoantibodies. CAART has been used in autoimmune neurological and dermatological diseases, targeting anti-muscle specific tyrosine kinase (MuSK) and anti-desmoglein 3 (DSG3) autoantibodies, and related clinical trials are ongoing in anti-MuSK antibody positive myasthenia gravis and anti-DSG3 antibody positive mucosal-dominant pemphigus vulgaris (NCT05451212, NCT04422912).

New generations of CAR are characterized by a combination of co-stimulatory domains or additional domains activating different intracellular signalling pathways, which aims to strengthen and prolong the expected effect of CAR T cells *in vivo*. One trial registered (NCT05459870) claimed to apply 4SCAR T cells, which is the fourth generation supposed to combine three co-stimulatory motifs as the intracellular domain. Similar products based on 4SCAR-T from the same institute have been reported safe and well tolerated in glioblastoma and paediatric neuroblastoma [54, 55].

Other cutting-edge updates are emerging to engineer safer and more effective CAR immune cells [56, 57]. Other than virus transduction, gene editing, especially mRNA technology, is another way to synthesize CAR T cells [58]. T cell-targeted lipid nanoparticles delivering modified mRNA was previously reported to treat cardiac injury *in vivo* [59]. Recently, Granit *et al.* [60] reported the phase 1b/2a result of a prospective, multicentre, open-label, non-randomized study that uses RNA-engineered BCMA-targeted CAR T cells in myasthenia gravis. Unlike conventional DNA-engineered CAR T cells, RNA-engineered CAR T cells do not persist long term and do not require lymphodepletion. The treatment was well tolerated with no adverse events typically associated with DNA-based CAR T cells (such as CRS) reported.

Gene editing technique also makes allogeneic CAR-T therapy possible [61] (Fig. 2). Allogeneic T cells could shorten the waiting time for infusion and reduce the cost of treatment, but it may cause life-threatening graft-*vs*-host disease (GVHD), and may be rejected by host immune systems [62]. Gene editing eliminates TCR expression and makes allogeneic CAR T cells undetectable by the host immune system, and thus could control the risk of GVHD. Except gene editing, choosing an appropriate cell source can also reduce the occurrence of GVHD. Although there are still challenges to be resolved in allogeneic CAR therapy, some products generated by universal platforms have entered clinical trial, including in ARDs (NCT05859997). Besides, CARs can be synthesized not only on the surface of T cells, but also on NK cells [63, 64], Tregs [65] or even macrophages (Fig. 2) [66]. CARs based on different cells can have different functional characteristics.

Although reported applications in ARDs are all in adult patient, CAR therapy has proven well tolerated in children [67]. Due to the chronic relapsing nature of ARDs, paediatric patients usually have higher cumulative exposure to glucocorticoids and other immunosuppressants than adults. It is encouraging to note that numerous ongoing clinical studies have included paediatric patients (Table 3).

## Conclusion

In conclusion, CD19-targeted CAR-T therapy is rapidly emerging as a potential treatment option for autoimmune rheumatic diseases. The success of future clinical trials hinges on designing CAR-based immune cells tailored according to the pathogenesis of various ARDs. Utilizing appropriate CAR engineering technology to minimize toxicity and maximize therapeutic efficacy is of paramount importance. Equally critical is the judicious selection of suitable subject populations for trials.

We maintain an optimistic yet cautious outlook on the application of CAR immune cell therapy in ARDs, and planned clinical trials will shed further light on efficacy and safety, thereby consolidating its potential as a promising future therapeutic strategy for ARDs.

## Data Availability

The data underlying this article are available in the article.
